# Electrochemical preparation of Al- Li alloy from Urea-LiCl molten salt at 353–393 K

**DOI:** 10.3389/fchem.2023.1159780

**Published:** 2023-03-23

**Authors:** Bingliang Gao, Zhiwei Liu, Yifan Deng

**Affiliations:** School of Metallurgy, Northeastern University, Shenyang, China

**Keywords:** lithium, Al-Li alloy, electrodeposition, room-temperature molten salt, urea

## Abstract

Al-Li alloy was prepared by electrodeposition on solid aluminum electrode in a urea-LiCl (77.5:22.5, mol%) molten salt system at 353–393 K. The results of cyclic voltammetry and chronopotentiometry show that underpotential deposition of Li occurs on the Al electrode and forms phases of Al(α) and AlLi(δ) which are confirmed by XRD measurements. Scanning electron microscopy observation of deposits indicates that Al-Li alloy with the thickness of 143 µm was obtained by potentiostatic electrolysis at −0.5 V vs. Li^+^/Li. The Li content in the alloy obtained at 373 K can reach 6.9 wt%. Galvanostatic electrolysis at 20 A demonstrated that Al-Li alloy coating on aluminum plate can be obtained from urea-LiCl electrolyte at 373 K in the atmosphere.

## 1 Introduction

Al-Li alloy is widely applied in aerospace, military industry and other fields because of low density, high elastic modulus, high strength, high specific stiffness, good corrosion resistance, etc. The typical Li content in the commercial Al-Li alloys is between 1-2 wt%. Higher Li contents result in poor ductility and fatigue performance (Dai et al., 2012). At present, most Al-based alloys are prepared by directly mixing and fusing the metallic elements, which has many drawbacks such as metal burning, high energy consumption and complex processing for achieving homogeneous alloy composition and refined grain size. Alternatively, some new methods have been suggested for preparation of Al-base alloys such as molten salt electrolysis (Cox and Fray, 2002), electrodeposition in room temperature ionic liquid (Morimitsu et al., 2000) and mechanical alloying (Suryanarayana, 2001), etc., During the smelting process of Al-Li alloys, lithium is prone to react with oxygen, water vapor, nitrogen in the air environment and hydrogen in the melt, resulting in an increase in the content of impurities in the alloy. During the casting process of Al-Li alloys, the big density difference between Al and Li can easily lead to alloy element segregation, uneven composition, and cracking. On the other hand, the risk of explosion of aluminum-lithium alloy melt caused by the reaction with the coolant is higher than other traditional aluminum alloys, and the explosion energy increases exponentially with the increase of lithium content. The above disadvantages lead to complex production process and high production cost of Al-Li alloy, which limits the large-scale application of Al-Li alloy.

It is very necessary to develop a low-energy, green, and simple process to prepare Al-Li alloys. Molten salt electrolysis has been tried for the preparation of Al-Li alloys with uniform composition and structure. Watanabe et al. prepared an Al-Li alloy with Li content of 19.1 wt% and Na, K, Ca content of less than 5 ppm by electrodeposition on solid aluminum electrode in the LiCl- KCl system (Watanabe et al., 1987). Gao, et al. prepared the Al-Cu-Li alloy by electro-deoxidation by using Al-Cu-Li_2_O as the cathode in the LiCl-KCl system at 648 K. The Li content in the alloy can reach up to 9.0 wt% (Gao et al., 2016).

In recent years, the rapid development of room-temperature molten salts has provided the possibility for the electrodeposition of Metal-Li alloys at room-temperature. Lithium, with the most negative electrochemical potential in electrochemical series, requires the supporting electrolyte/solvent with high electrochemical stability to ensure the preferential reduction of Li(I). Yang, *et al.* used LiBF_4_ as lithium source in 1-butyl-3-methylimidazolium tetrafluoroborate [(BMIm) (BF_4_)] ionic liquid, and obtained a uniform, bright and adherent Cu-Li alloy coating on copper electrode (Yang et al., 2013). However, imidazole-based ionic liquids are expensive and not air/moisture stable, which is a key problem for realization of industrial production. As a cheap organic compound and easy to produce on a large scale, urea can form low-melting molten salts with alkali metal halides characterized with high conductivity and wide electrochemical window (Liang et al., 2001; Liu et al., 2017). At present, there are a few reports on the preparation of active metals and their alloys by electrodeposition in urea-based room temperature molten salts (Liu et al., 2006; Wang et al., 2008; Li et al., 2009).

In this paper, the electrochemical formation of Al-Li alloy on aluminum electrode was investigated in urea-LiCl low-temperature molten salt in range 353–393 K. The melt structure of urea-LiCl (77.5:22.5, mol%) was studied by Raman spectroscopy. The electrochemical behavior of Li in urea-LiCl molten salt on Al electrode was studied by cyclic voltammetry and chronopotentiometry. The effect of temperature on the quality of deposits was investigated with the assistance of XRD, SEM and ICP-AES.

## 2 Experimental

### 2.1 Preparation and purification of the melt

Urea (Sinopharm Group Reagent Co., Ltd. 99% purity), and LiCl (Shanghai Aladdin Biochemical Technology Co., Ltd. 99% purity) were vacuum-dried at 393K and 473 K for 72 h, respectively, and stored in a glove box (MB-200B, MBRAUN, Gremany) filled with high-purity argon. The water and oxygen contents were maintained below 0.1 ppm in the glove box. The urea-LiCl mixture (77.5:22.5, mol%) was melted at 353 K and stirred slowly until a uniform, colorless and transparent electrolyte was formed.

### 2.2 Electrochemical apparatus and electrodes

All electrochemical tests, such as cyclic voltammetry, chronopotentiometry and potentiostatic electrolysis were carried out using an electrochemical workstation (CHI660E, Shanghai Chenhua Instrument Co., Ltd.) in the glove box. Cyclic voltammetry and chronopotentiometry were performed in a three-electrode electrochemical cell. The working electrode is an aluminum wire (99.99% purity, d = 0.7 mm, active area 0.18 cm^2^), which was polished with sandpaper, then ultrasonically cleaned in acetone and absolute ethanol, and then air-dried. The reference electrode and the counter electrode are lithium sheets (99.9% purity).

### 2.3 Preparation and analysis of Al-Li alloy deposit

Al-Li alloy samples were prepared by potentiostatic electrolysis at an aluminum sheet (99.99% purity, 10 mm × 10 mm × 0.3 mm). After electrodeposition, the sample was cleaned by ethylene carbonate solvent to remove adhered molten salts at 353K, and then vacuum-dried at 353 K for 24 h. The sample was analyzed by XRD (MPDDY 2094, PANalytical, Netherlands, Cu-K *α* line, 40 kV 40 mA). To avoid the influence of moisture on the sample during the transfer and detection process, the sample was sealed on the glass slide with a parafilm in the glove box. SEM (EVO18, ZEISS, Germany) was used to observe the surface morphology and the thickness of the deposit. The lithium content in the alloy was analyzed by an inductively coupled plasma atomic emission spectrometer (ICP—AES, Optima 8300DV, PE, America). The standard deviation of ICP-AES for determination of Li content in Al-Li alloy is less than 5%.

A 20A scale electrolysis experiment was carried out in the atmosphere. 2.4 kg electrolyte was melted in a 3,000 mL beaker. The cathode is an aluminum plate with dimensions of 125 mm × 180 mm×2 mm. The anode is a graphite plate with dimensions of 100 mm × 180 mm×4 mm. Three anodes and two cathodes were arranged in series with anode-cathode distance of 20 mm in the electrolysis cell. The melt temperature was controlled at 373 K. The beaker was sealed with a rubber plug. The argon gas was input into the electrolyte for bubbling the melt. After 1 h at constant current of 20 A, the cathodes were cleaned by ethylene carbonate solvent to remove adhered molten salts at 353 K, and then vacuum-dried at 353 K for 24 h.

### 2.4 Characterization of the melt structure

The molten urea-LiCl electrolyte was sealed in a L-shape quartz tube at 353 K, and then analyzed at the same temperature using a Raman spectrometer (HR800, Horiba Jobin Yvon, France). The applied He-Ne laser was excited at a wavelength of 633 nm.

## 3 Results and discussion

### 3.1 Raman spectrum of urea-LiCl molten salt

**FIGURE 1:**
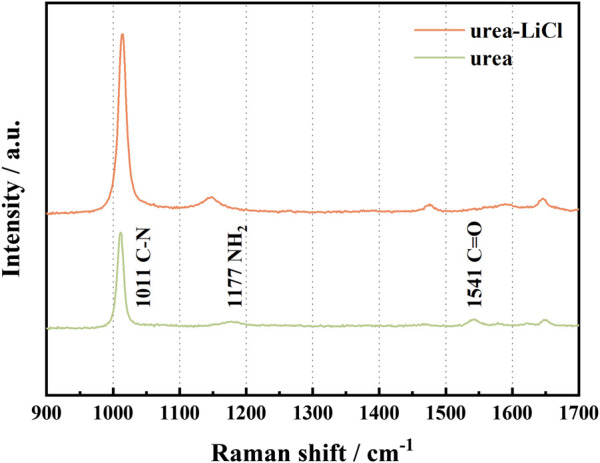
Raman spectra before and after adding LiCl in Urea.

The Raman spectra of Urea before and after adding LiCl are shown in Figure 1. In the range of 900–1700 cm^−1^, the addition of LiCl results in an obvious blue shift of the stretching vibration of C=O (1,541 cm^−1^), which is due to the formation of coordination compounds between Li ^+^ and urea (Theophanides and Harvey, 1987; Keuleers et al., 1999). At the same time, the formation of this coordination bond can weaken or even destroy the hydrogen bond existing in urea (Liang et al., 2001). On the other hand, Cl^−^ connects with urea through hydrogen bonds, which causes the blue shift of NH_2_ vibration (1,177 cm^−1^, rocking vibration in the symmetry plane). The interaction between Urea and LiCl causes the broadening of the Raman scattering peak corresponding to the CN vibration (1,011 cm^−1^, symmetric stretching vibration). In summary, the formation of the coordination bond between Li^+^ and C=O, and the hydrogen bond between Cl^−^ and urea weakens the ionic bond between Li^+^ and Cl^−^, which is beneficial to the separation of anions and cations in LiCl, forming urea-LiCl low-temperature molten salt electrolyte as a result.

### 3.2 Electrochemical behavior of urea -LiCl molten salt on the Al electrode

**FIGURE 2:**
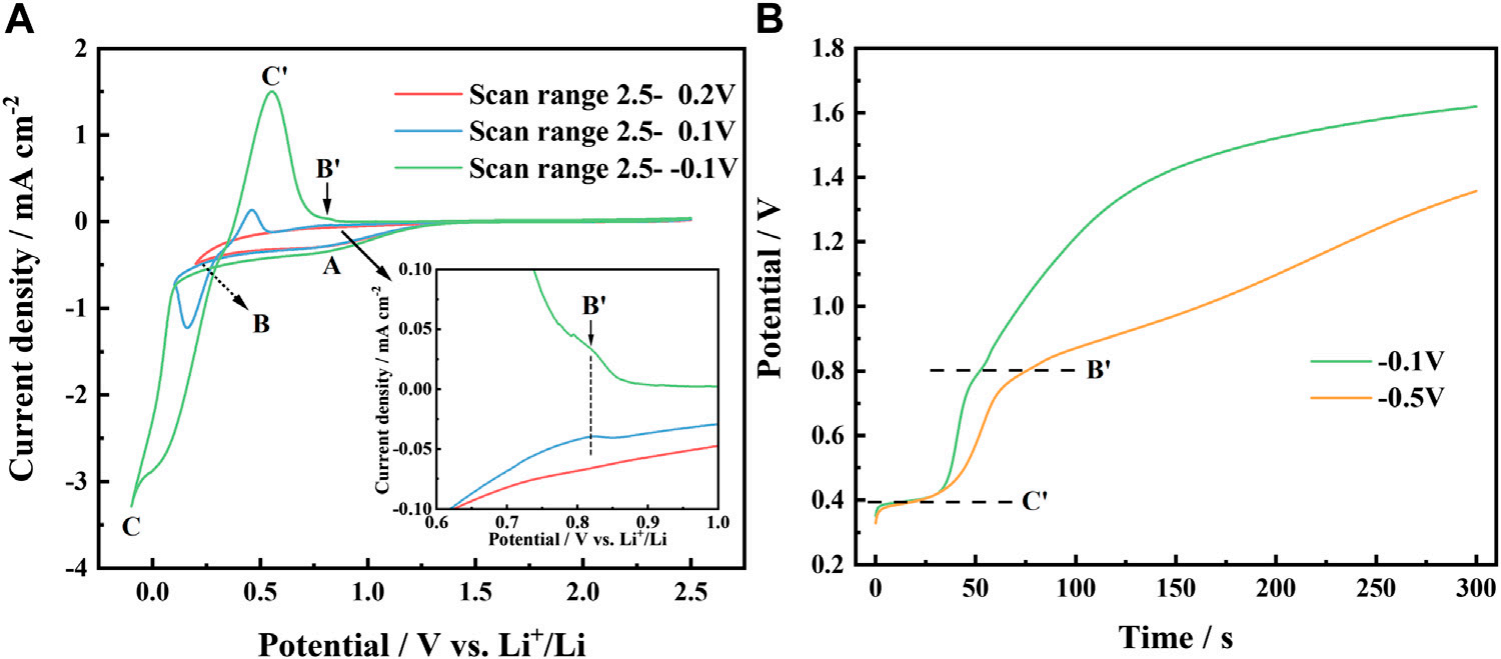
**(A)** Cyclic voltammetry curves of urea -LiCl molten salt on Al electrode with various reverse potentials at 353 K, scan rate 50 mV/s; **(B)** Evolution of the chronopotential curves of deposits on Al electrode in urea -LiCl molten salt at various potentials for 30 s at 353 K.

Figure 2A presents the effect of decreasing the reverse potential on the cyclic voltammetry curves of urea-LiCl molten salt on the Al electrode at a scan rate of 50 mV/s at 353 K. In all curves, a reduction wave A is observed to start at approximately 1.3 V (vs. Li^+^/Li). There is no obvious oxidation current in the reverse scan at more positive potential. It indicates that wave A might be ascribed to the reduction of hydrogen, which reaction scheme can be described by the Eq. 1 (Liu et al., 2006; Wang et al., 2008; Li et al., 2009; Ogawa and Mori, 2020).

$$R-CO-{NH}_{2}+e\to 1/2H_{2}+R-CO-{NH}^{-}\left(R={NH}_{2},{CH}_{3}\right)$$ (1)

If the reverse potential was set at a more negative value, a current loop could be observed (see blue curve and green curve in Figure 2A), which is a typical symptom of metal nucleation process. Obviously, the reduction peak C appeared at 0.16 V, and its corresponding oxidation peak C ′ appeared at 0.46 V, can be ascribed to the deposition and dissolution of Li, respectively. The reduction peak potential of Li is obviously positive than 0 V (vs. Li^+^/Li), which is the result of the underpotential deposition of Li on Al to form Al-Li alloy.

In the cathodic scans starting from approximately 0.5 V, a new reduction wave B is observed. In the potential range from 0.6 V to 1.0 V in the anodic scans, the partial enlarged view (Figure 2A inset) shows a small oxidation shoulder B′, which becomes more significant if the reverse potential was set at a more negative potential. It indicates that the reduction of Li actually occurs when potential moves to a negative value less than 0.5 V. The reduced lithium atoms diffuse into the matrix of aluminum and form *α*-Al phase on the surface layer of the cathode. To confirm this, the chronopotential curves were recorded after potentiostatic electrodeposition at −0.1 V and −0.5 V for 30 s, respectively, as shown in Figure 2B. It illustrates that the deposit proceeded a two-stage dissolution process at 0.4 V and 0.8 V, respectively, which is consistent with the cyclic voltammetry test results. Such electrochemical behavior of Li in urea-based melt is quite different from that of the LiBr-KBr-CsBr eutectic melt at 523–673 K, presented by Kasajima et al. (2004). He observed three potential plateaus at 0.02 V, 0.075 V and 0.299 V, and assigning them to (AlLi(γ)+Liq.) (AlLi(δ)+AlLi(γ)) (Al(α)+AlLi(δ)), respectively, in the open-circuit potentiometry. In our investigation, the electrodeposition was carried out at temperatures below the melting point of lithium. At lower temperature, the mobility of lithium ions in urea-based melt is also slower than that of high temperature melt. Consequently, the depositing rate of lithium is significantly slower as well as diffusion rate of lithium into the matrix of aluminum in urea-LiCl melt. It is rational that different phase composition and electrochemical behavior are presented. Based on the above electrochemical test results combined with the Al-Li alloy phase diagram (Liu et al., 2021), it is believed that two Al-Li alloy phases were formed during the deposition process, and correspondingly two oxidation peaks on the anodic branch during the dissolution process.

### 3.3 Potentiostatic electrodeposition

**FIGURE 3:**
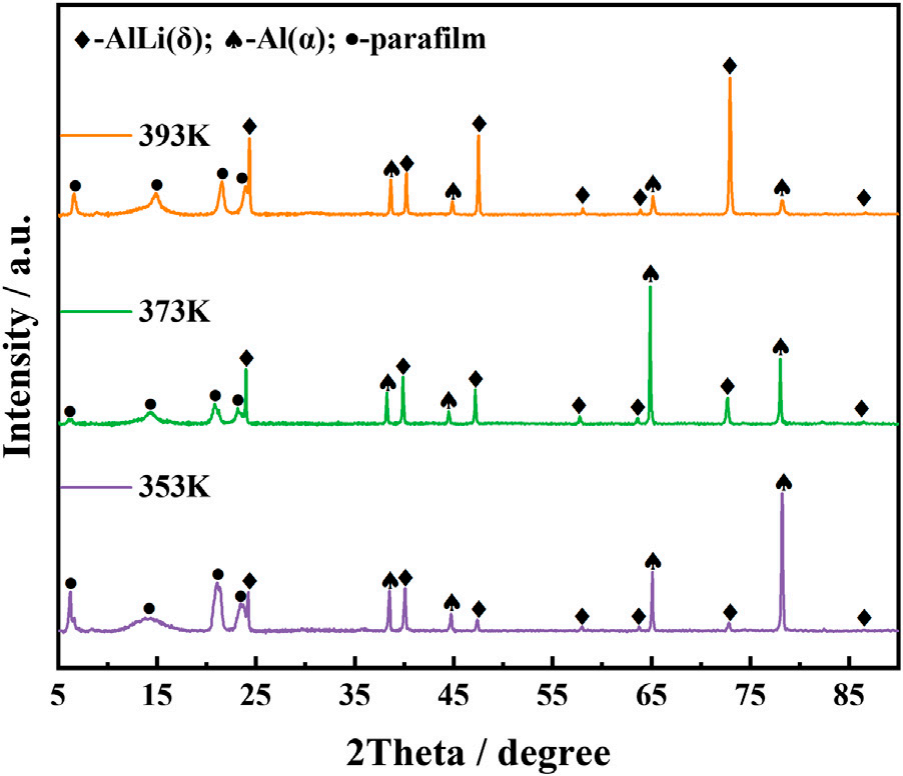
XRD patterns of Al-Li alloy samples obtained by electrodeposition at −0.5 V al for 1 h at various temperatures (353 K, 373 K, 393 K).

To obtain thick layer of Al-Li alloy on the surface of aluminum plate, and suppress the side reaction of hydrogen generation, the potentiostatic electrodeposition was carried out at −0.5 V for 1 h at 353 K, 373 K, 393 K, respectively. Figure 3 presents the XRD patterns of the products. The phases were identified as mixture of *α*-Al and AlLi(δ). The surface of the Al electrode has transformed into Al-Li alloys. Li content increased remarkably from 1.2 wt% to 6.9 wt% when temperature was increased from 353 K to 373 K. *α*-Al shares the same fcc crystal structure with aluminum, therefore their XRD diffraction patterns are almost same except for the difference in peak intensities. According to Liu’s investigation on Al-Li phase diagram by means of first principles calculations, Al_3_Li, a metastable phase, should exist in the fcc structure at room temperature (Liu et al., 2021), which also shares the similar XRD patterns with aluminum. Compared to deposit obtained at 353 K, some peak intensities change in the XRD patterns of deposit prepared at 373 K, such as increase of 2-theta angle at 65, decrease of 2-theta angle at 78. It might be an indication of phase change from *α*-Al to Al_3_Li. The energy barrier for the transformation of Al_3_Li into AlLi(δ) is very small and can be overcome by thermal vibrations (Liu et al., 2021). Obviously, higher temperature will promote the transformation of Al_3_Li to AlLi(δ). Although Li content (6.8 wt%) in deposit obtained at 393 K does not increase compared to deposit at 373 K, the content of AlLi(δ) phase increased remarkably as shown in Figure 3. However, no stand evidence for existence of Al_3_Li phase can be obtained in this paper, all XRD patterns relating to Al, *α*-Al and Al_3_Li were marked as *α*-Al in the Figure 3.

At a potential of about 0.5 V, Li starts to deposit on the fresh Al electrode and forms the *α*-Al phase (Fung et al., 1982), which is a solid solution in the aluminum-rich side according to the Al-Li alloy phase diagram (Liu et al., 2021). Since the atomic radii of Li and Al are close (Li, 0.155 nm; Al, 0.138 nm), aluminum and lithium atoms share the fcc lattice. With the increase of Li content, as shown in CVs in Figure 2A with the reverse potential as 0.1 V (blue line) and −0.1 V (green line), respectively, a new alloy phase, AlLi(δ) phase was formed. Compared with *α*-Al phase, AlLi(δ) phase has a more open structure (bcc, a = 0.637 nm) (James, 1976), which promotes Li deposition and alloying. These results proves that the potential plateau at 0.8 V in Figure 2B corresponds to the phase *α*-Al(Li), and the potential plateau at 0.4 V corresponds to the coexisting phase of *α*-Al(Li)+ AlLi(δ).

**FIGURE 4:**
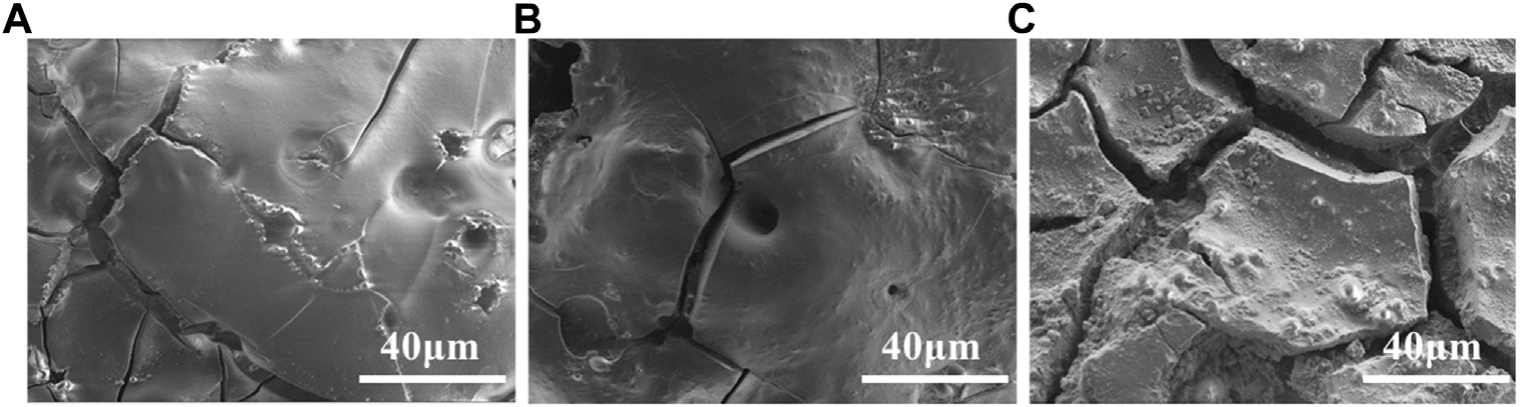
SEM photos of Al-Li alloy samples obtained by potentiostatic electrodeposition at −0.5 V for 1 h at various temperatures: **(A)** −353 K; **(B)** −373 K; **(C)** −393 K.

SEM microscopic characterization shows (see Figure 4) that the surface of the alloy is uneven and rough with many holes and cracks. The surface roughness of the alloy increases with the electrolysis temperature, which is related to the AlLi(δ) content in the deposit. With increasing of Li content and temperature, the fcc crystal structure of aluminum electrode is gradually transformed into bcc structure of Al-Li alloy. The volume change caused by the phase transformation leads to the generation of internal stress, which causes alloy deformation and cracking.

**FIGURE 5:**
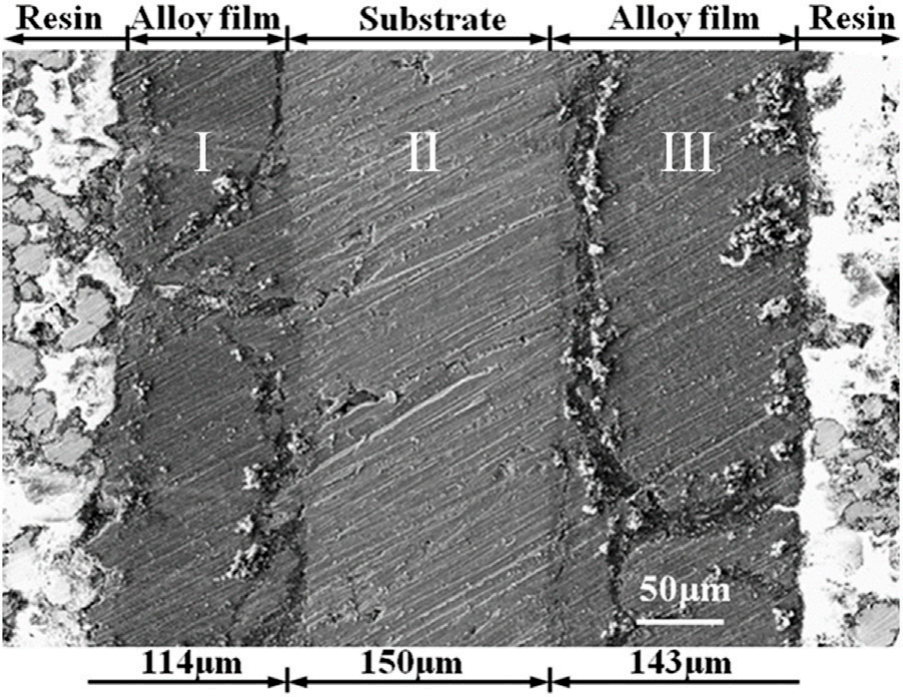
SEM cross-sectional photograph of Al-Li alloy sample obtained by potentiostatic electrodeposition at −0.5 V for 1 h at 373 K.

From the cross-sectional structure of the electrode (Figure 5), the electrode is divided into three layers: light layer of aluminum substrate and dark layers of Al-Li alloy. The total thickness of the electrode increased from 300 µm before the deposition to about 400 μm after the deposition. The thicknesses of the Al-Li alloy layer (I) and the Al-Li alloy layer (III) is 114 μm (I) and 143 μm (III), respectively. The aluminum plate cathode was set between two anodes. However, we tried to set the anode to cathode distance evenly in two compartments of the cell. Considering the low conductivity of urea-LiCl melt at near room temperature, the tiny difference in anode to cathode distance might cause uneven current distribution. Consequently, the thickness of deposit on each side of the cathode might be different.

### 3.4 20A galvanostatic electrolysis in the atmosphere

**FIGURE 6:**
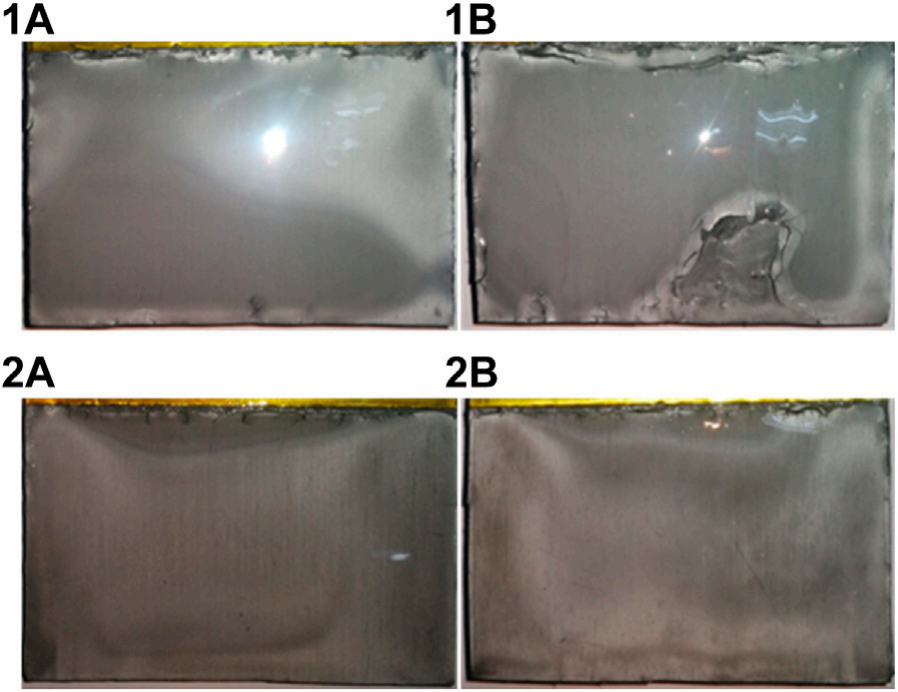
Optical photos of Al-Li alloy deposits obtained from urea-LiCl by Galvanostatic electrolysis at 20 A for 1 h at 373 K. 1A and 2A are two sides of the cathode 1; 1B and 2B are two sides of the cathode 2.

For industrial application, it is meaningful to carry out electrolysis in the atmosphere without need for a glove box. Figure 6 shows the deposits obtained in a 20 A scale electrolysis from the melt of urea-LiCl at 373 K. The electrodeposition was proceeded on both sides of two cathodes. The deposits were analyzed with XRD, which confirmed that the dominant crystal structures were as same as that shown in Figure 3. The thickness of the deposits is in the range from 100 μm to 170 μm.

## 4 Conclusion

It is feasible to electrochemically prepare Al-Li alloys in the low-temperature molten salt electrolyte of urea- LiCl. Cyclic voltammetry test and chronopotential test results show that Li^+^ starts to reduce on the surface of Al electrode to form *α*-Al phase at 0.5 V (vs. Li^+^/Li), and form AlLi(δ) phase at 0.16 V. Potentiostatic electrolysis confirmed that the phase composition and lithium content could be controlled by adjusting electrolysis temperature. The paper provides us a possibility for surface modification of aluminum-based material by formation of Al-Li coating electrochemically in the melt of urea-LiCl at lower temperature.

## Data Availability

The raw data supporting the conclusion of this article will be made available by the authors, without undue reservation.
